# Biopsy-proven kidney involvement in hypocomplementemic urticarial vasculitis

**DOI:** 10.1186/s12882-022-02689-8

**Published:** 2022-02-16

**Authors:** Alice Corthier, Marie Jachiet, Daniel Bertin, Aude Servais, Christelle Barbet, Adrien Bigot, Marie-Sylvie Doutre, Didier Bessis, Ancuta Bouffandeau, Olivier Moranne, Pierre-André Jarrot, Nathalie Bardin, Benjamin Terrier, Stephane Burtey, Xavier Puéchal, Laurent Daniel, Noémie Jourde-Chiche

**Affiliations:** 1 Department of Nephrology, Aix-Marseille Univ, Hôpital de la Conception, AP-HM, 147 Bd Baille, 13005 Marseille, France; 2 Department of Dermatology, Hôpital Saint Louis, AP-HP, Université Paris Diderot, Paris, France; 3 Laboratory of Immunology, Hôpital de la Conception, AP-HM, Marseille, France; 4 Department of Nephrology, Hôpital Necker, AP-HP, Paris, France; 5 Department of Nephrology, CHU de Tours, Tours, France; 6 Department of Internal Medicine, CHU de Tours, Tours, France; 7 Department of Dermatology, CHU de Bordeaux, Bordeaux, France; 8 Department of Dermatology, Hôpital Saint Eloi, Université de Montpellier, Montpellier, France; 9 Department of Nephrology, CH d’Evreux, Evreux, France; 10 Department of Nephrology, Dialysis, Apheresis, CHU Caremeau, Nimes, France; 11 Université de Montpellier-Nîmes, Nîmes, France; 12 Department of Internal Medicine and Clinical Immunology, Hôpital de la Conception, AP-HM, Marseille, France; 13 Aix-Marseille Univ, C2VN, INSERM 1263, INRA 1260, Marseille, France; 14 Department of Internal Medicine, National Reference Center for Rare Systemic Autoimmune Diseases, Cochin Hospital, Paris, France; 15 Paris Descartes University, Paris, France; 16 Department of Pathology, Aix-Marseille Univ, Hôpital de la Timone, AP-HM, Marseille, France

**Keywords:** Hypocomplementemic urticarial vasculitis, McDuffie syndrome, Glomerulonephritis, Renal vasculitis, Renal biopsy, Anti-C1q antibody, C1q deposits

## Abstract

**Background:**

Hypocomplementemic urticarial vasculitis (HUV) is a rare systemic vasculitis. We aimed to describe the kidney involvement of HUV in a multicenter national cohort with an extended follow-up.

**Methods:**

All patients with HUV (international Schwartz criteria) with a biopsy-proven kidney involvement, identified through a survey of the French Vasculitis Study Group (FVSG), were included. A systematic literature review on kidney involvement of HUV was performed.

**Results:**

Twelve patients were included, among whom 8 had positive anti-C1q antibodies. All presented with proteinuria, from mild to nephrotic, and 8 displayed acute kidney injury (AKI), requiring temporary haemodialysis in 2. Kidney biopsy showed membrano-proliferative glomerulonephritis (MPGN) in 8 patients, pauci-immune crescentic GN or necrotizing vasculitis in 3 patients (with a mild to severe interstitial inflammation), and an isolated interstitial nephritis in 1 patient. C1q deposits were observed in the glomeruli (*n* = 6), tubules (*n* = 4) or renal arterioles (*n* = 3) of 8 patients. All patients received corticosteroids, and 9 were also treated with immunosuppressants or apheresis. After a mean follow-up of 8.9 years, 6 patients had a preserved renal function, but 2 patients had developed stage 3–4 chronic kidney disease (CKD) and 4 patients had reached end-stage kidney disease (ESKD), among whom 1 had received a kidney transplant.

**Conclusion:**

Renal involvement of HUV can be responsible for severe AKI, CKD and ESRD. It is not always associated with circulating anti-C1q antibodies. Kidney biopsy shows mostly MPGN or crescentic GN, with frequent C1q deposits in the glomeruli, tubules or arterioles.

**Supplementary Information:**

The online version contains supplementary material available at 10.1186/s12882-022-02689-8.

## Key messages

- Kidney involvement in HUV can be life-threatening and lead to end-stage kidney disease.
- Glomerular, vascular and tubulo-interstitial compartments can be injured, with frequent C1q deposits.
- Systematic screening for kidney involvement during flares of HUV could accelerate diagnosis and treatment and reduce kidney damage.

## Introduction

Hypocomplementemic urticarial vasculitis (HUV) is a rare systemic vasculitis, also called “McDuffie syndrome” or “anti-C1q vasculitis”. It was classified among immune complex small vessel vasculitides in the revised international Chapel Hill consensus conference in 2012 [1]. It was first described by McDuffie et al. in 1973 and then well defined in 1982 by Schwartz et al. [2, 3]. They proposed diagnostic criteria composed of 2 major criteria (recurrent urticarial lesions for at least 6 months and hypocomplementemia) associated with 2 minor criteria among the following: leukocytoclastic vasculitis (on cutaneous biopsy), arthralgia or arthritis, glomerulonephritis, ocular inflammation (uveitis or episcleritis), recurrent abdominal pain or anti-C1q antibody positivity. The frequency of kidney involvement associated with HUV ranges from 14 to 50% across different cohorts, and is poorly defined [4–6]. In the French national cohort published in 2015 by Jachiet et al. [5], a kidney involvement of HUV was observed in 10 (18%) patients, but detailed pathological lesions were not available. In addition, little is known on the long-term prognosis of kidney involvement in HUV, and no specific therapeutic strategy is defined in the literature. We describe here the clinical presentation, the pathological lesions and the therapeutic management of a multicentre cohort of 12 patients with biopsy-proven kidney involvement associated with HUV, identified through the French Vasculitis Study Group (FVSG) network.

## Patients and methods

This is a multicentre retrospective study. All cases of patients with HUV, as defined by Schwartz criteria, and a biopsy-proven kidney involvement, identified through a survey sent to physicians from the French Study Group on Vasculitis (FSGV) network, were reviewed and included. Some of these patients were included in the national cohort by Jachiet et al. The following data was collected for each patient: demographic characteristics, systemic symptoms, skin lesions, adenopathy, rheumatic, ocular, eyes nose throat (ENT), lung, gastrointestinal, neurologic, cardiac involvement, and detailed renal parameters. AKI was defined according to the RIFLE criteria as R (risk), I (injury) and F (failure) [7]. Serum levels of C-reactive protein (CRP), complement fractions C3 and C4, anti-C1q antibodies, antinuclear antibodies (ANA), antiphospholipid antibodies, rheumatoid factor, cryoglobulinemia, anti-neutrophil cytoplasmic antibodies (ANCA) and protein electrophoresis were collected. All patients were tested for immunization against hepatitis B, C, HIV and syphilis. Results of skin biopsies were collected when available. All patients had at least one kidney biopsy, analysed by light microscopy and immunofluorescence. Electronic microscopy description was reported when available. The therapeutic strategies, global outcome and renal outcome were collected. Chronic kidney disease (CKD) was defined as a permanently altered kidney function with an estimated glomerular filtration rate (eGFR) < 60 mL/min/1.73m^2^ using the MDRD formula [8].

We then performed a systematic literature review on cases of HUV with renal involvement, using the following MESH terms: Hypocomplementemic urticarial vasculitis, Mac Duffie, kidney/renal involvement, acute kidney injury, chronic kidney disease, glomerulonephritis and kidney/renal biopsy.

For statistical analysis, continuous variables were compared using the Wilcoxon-Mann-Whitney test on mean or median values; categorical variables were compared using the Fisher’s exact test.

## Results

### Patients’ characteristics

Twelve patients with a biopsy-proven kidney involvement of HUV were identified, of whom 6 were previously included in the cohort of Jachiet et al. Their clinical and biological characteristics are detailed in Table 1. Two patients had a paediatric onset of HUV. Chronic urticarial lesions were present in all patients. Arthralgia were reported in 8 patients, with clinical arthritis in 3, and lymphadenopathies in 5 patients. Five patients displayed ocular involvement of HUV: anterior uveitis in 3 patients, conjunctivitis in 2, retinal involvement in two. Pulmonary symptoms (recurrent bronchitis) were described by 3 patients, among whom 1 had chronic respiratory failure. One patient displayed a pericarditis. Anti-C1q antibodies were positive in 8 patients. ANA were detected at some point during the follow-up in 7 patients, among whom one had anti-SSA and anti-double stranded DNA antibodies (patient #12), and another had anti-SSA antibodies and positive cryoglobulinemia (patient #7). ANCA antibodies were detected in 6 patients at some point during the follow-up, among whom 3 had anti-myeloperoxidase (MPO) antibodies, 1 anti-proteinase 3 (PR3) antibodies, and 2 no antigenic specificity. There was no difference in the characteristics of patients with or without ANCA, or with or without ANA, except for the higher frequency of ANA positivity in patients with ANCA, and of ANCA positivity in patients with ANA (Supplementary Tables 1 and 2).

**Table 1:** Clinical and biological presentation of the 12 patients from the present cohort

	Patient 1	Patient 2	Patient 3	Patient 4	Patient 5	Patient 6	Patient 7	Patient 8	Patient 9	Patient 10	Patient 11	Patient 12
Age at kidney involvement	42	12	41	40	41	5	44	56	55	76	45	43
Gender	F	F	F	F	F	M	F	F	F	M	F	F
**Extra-renal involvement**												
Asthenia/fever	+/+	+/+	+/−	+/−	+/−	−/+	+/−	−/−	−/−	+/+	+/+	+/−
Chronic urticaria	+	+	+	+	+	+	+	+	+	+	+	+
Angioedema/purpura/livedo	−/+/+	−/−/−	−/+/−	+/−/−	+/−/−	−/−/−	+/−/−	−/−/−	+/−/−	−/+/−	−/−/−	−/−/−
Cutaneous leucocytoclastic vasculitis	+	–	+	+	+	ND	–	+	ND	+	ND	+
Arthralgia/arthritis	+/+	+/+	+/−	−/−	+/+	−/−	+/−	−/−	+/−	+/−	−/−	+/−
Anterior uveitis/conjunctivitis	+/+	−/+	−/−	−/−	+/−	−/−	−/−	−/−	−/−	−/−	−/−	+/−
Retinopathy	+	–	–	–	–	–	–	+	–	–	–	–
Lymphadenopathy	+	–	–	–	+	–	+	–	+	–	–	+
PRESS/peripheral neuropathy	+/−	+/−	−/−	−/−	−/−	−/−	−/−	−/−	−/−	−/−	−/−	−/−
Respiratory disease	–	+	–	+	–	–	–	+	–	–	–	–
Pericarditis	+	–	–	–	–	–	–	–	–	–	–	–
**Renal presentation**												
Time since first symptoms of HUV (months)	84	22	37	55	49	42	60	0,5	36	76	0	6
Proteinuria	+	+	+	+	+	+	+	+	+	+	+	+
Nephrotic syndrome	+	–	+	+	–	+	+	–	+	–	–	–
Microscopic/macroscopic haematuria	+/−	−/−	+/+	+/+	−/−	+/+	+/+	+/−	+/+	+/−	−/−	+/−
Serum creatinine (μmol/L)	225	703	325	90	71	ND	160	77	326	608	268	140
AKI	+	+	+	–	–	–	+	–	+	+	+	+
Hemodialysis at diagnosis	–	+	–	–	–	–	–	–	–	+	–	–
High blood pressure	+	+	–	–	–	–	+	+	+	+	–	+
Pathological diagnosis	MPGN	MPGN	MPGN	MPGN	MPGN	MPGN	MPGN	MPGN	PI CGN	PI CGN	NV	IN
**Biology**												
Elevated CRP	+	ND	ND	ND	ND	ND	ND	–	+	+	+	+
Low C3	+	+	+	+	+	+	+	+	ND	+	ND	+
Low C4	+	+	+	+	+	+	+	+	+	+	ND	+
Low CH50	+	+	+	+	+	+	ND	+	ND	+	ND	+
Low C1q	+	+	+	ND	+	ND	ND	ND	+	+	ND	+
Anti-C1q antibody	+	ND	+	–	+	ND	+	+	+	–	+	+
Antinuclear antibody	–	+	–	+	–	–	+	+	+	–	+	+
Antiphospholipid antibody	–	–	ND	–	ND	ND	ND	ND	ND	–	+	ND
Rheumatoid factor	+	–	–	–	–	–	–	ND	ND	–	–	+
Cryoglobulinemia	+	–	–	–	–	–	+	+	ND	+	–	+
ANCA (anti-MPO/anti-PR3)	–	+ (+/−)	–	–	–	–	+ (+/−)	+ (−/+)	+ (+/−)	–	+ (−/−)	+ (−/−)
Monoclonal gammapathy	–	–	–	–	–	–	–	–	–	+	–	–

*PRESS* Posterior reversible encephalopathy syndrome, *AKI* acute kidney injury, *CRP* C-reactive protein, *ANCA* anti-neutrophil cytoplasmic antibodies, *F* female, *M* male, *ND* not determinate, + positive, − negative, *MPGN* membrano proliferative glomerulo nephritis, *PI CGN* pauci-immune crescentic glomerulonephritis, *NV* necrotizing vasculitis, *IN* interstitial nephritis

### Renal involvement of HUV

**Fig. 1:**
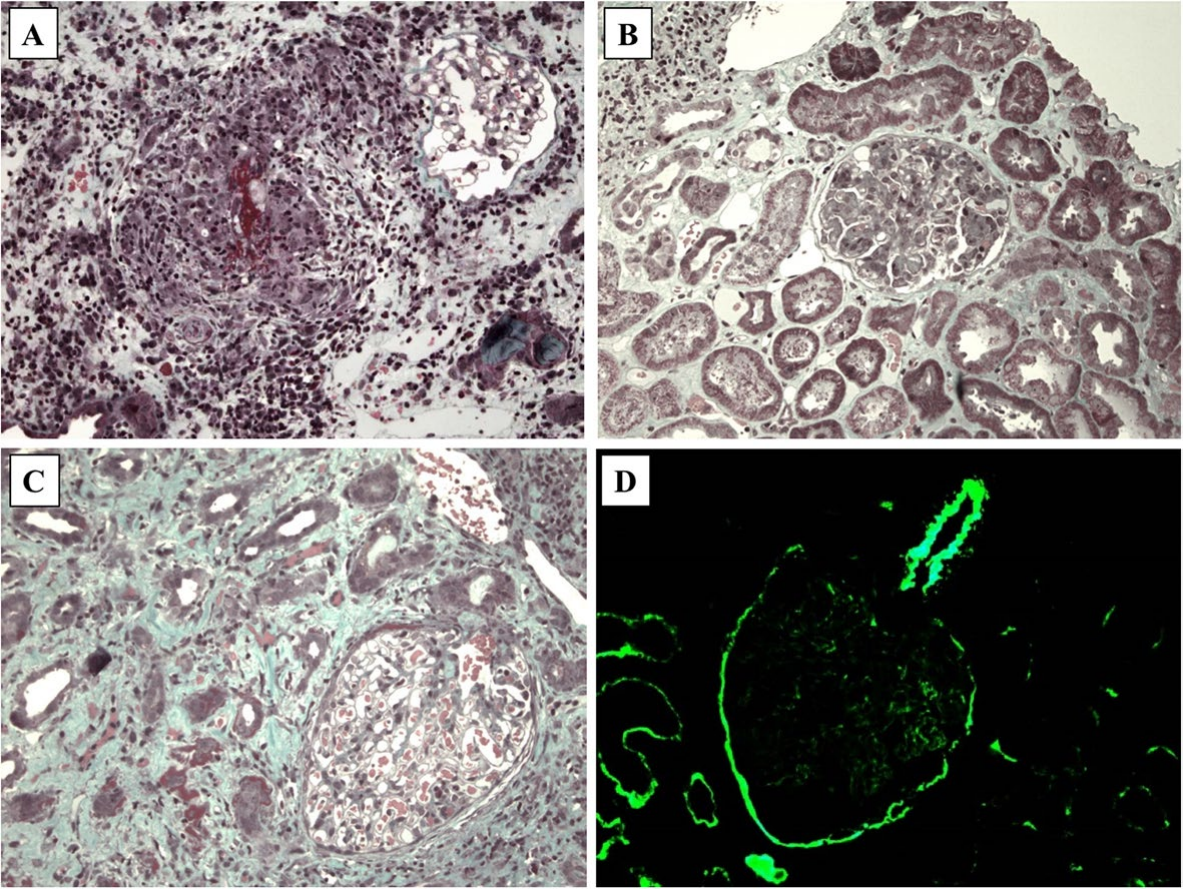
Representative kidney pathological lesions observed in patients with Hypocomplementemic Urticarial Vasculitis from the present cohort. **A** Vasculitis with fibrinoid necrosis involving a pre-glomerular arteriole. Masson’s trichrome, × 200. **B** Global endocapillary proliferation with an interstitium containing a slight peri-venular inflammatory infiltrate. Masson’s trichrome, × 200. **C** Interstitial nephritis with mononuclear cells, oedema, and suffering tubules. Masson’s trichrome, × 200. **D** Abundant deposits of C1q within arteriole wall, capsule, and tubular basement membrane. Immunofluorescence, × 200

All patients presented with proteinuria, from mild to nephrotic. Eight patients presented with acute kidney injury (AKI), requiring temporary hemodialysis in 2 patients. One patient had stage R, 5 stage I and 3 stage F AKI. Pathological examination of kidney is detailed in Table 2 for the 12 patients, among whom 2 had a repeat biopsy. Kidney lesions were various, with different patterns of glomerular, vascular, and tubulo-interstitial involvements (Fig. 1). A membrano-proliferative GN (MPGN) was documented in 8 patients, with crescents in 7, and with a full-house immunofluorescence pattern in 1 patient. Four patients had active vascular lesions: 2 had a necrotizing vasculitis (patients #2 and #11), and 2 had immune deposits of IgG, IgM, C3 and C1q in vascular walls (patient #2 and #9). One patient (#12) had an isolated interstitial nephritis without glomerular or vascular changes. The repeat biopsy of patients #2 and #7 showed consistent glomerular and vascular patterns, with different levels of inflammatory and fibrotic lesions. Second biopsy of patient #9 showed severe chronic lesions and glomerulosclerosis. Electron microscopy analysis was performed in 2 patients. It showed, in patient #4, abundant and diffuse electron-dense segmental subepithelial and subendothelial granular deposits in capillary walls; in the second biopsy of patient #7, few glomerular electron-dense granular deposits.

**Table 2:** Kidney pathological findings in the 12 patients from the present cohort

	Patient 1	Patient 2 (2003)	Patient 2 (2008)	Patient 3	Patient 4	Patient 5	Patient 6	Patient 7 (2012)	Patient 7 (2016)	Patient 8	Patient 9 (2018)	Patient 9 (2021)	Patient 10	Patient 11	Patient 12
**Pathological diagnosis**	**MPGN**	**MPGN**	**MPGN**	**MPGN**	**MPGN**	**MPGN**	**MPGN**	**MPGN**	**MPGN**	**MPGN**	**Pauci-immune CGN**	**Advanced** **chronic lesions**	**Pauci-immune CGN**	**Necrotizing vasculitis**	**Interstitial nephritis**
Number of glomeruli	21	7	13	12	22	11	45	13	16	9	21	18	22	10	14
Endocapillary hypercellularity	severe	mild	mild	mild	moderate	mild	moderate	mild	mild	moderate	–	–	–	–	–
Crescents (% of glomeruli)	30	15	–	–	15	–	10	–	5	–	40	–	15	–	–
Glomerular sclerosis (%)	30	–	30	–	–	–	–	60	60	–	15	> 90	20	–	15
Subendothelial deposits	IgG+++ C3+/−	IgG+ IgM+/− C3+/− C1q+	IgG+ C3+	IgG++ IgM++ C3+++ C4++ C1q+++	IgG+ IgM+ C3++ C4+ C1q++	IgA+ IgG+ IgM+ C1q +	IgG++ C3+++	IgG+ IgM+ C3+++ C1q++	IgM++ C3++ C1q+	IgA+ IgG++ IgM+/− C3+/− C1q +/−	IgA+	–	–	–	–
Micro thrombi	+	–	–	+	+	–	–	–	–	–	–	–	–	–	–
Interstitial inflammation	severe	severe	mild	severe	mild	mild	mild	severe	severe	moderate	moderate	–	moderate	severe	moderate
Interstitial fibrosis	mild	–	moderate	–	–	mild	–	severe	severe	moderate	moderate	severe	mild	–	mild
Tubular immune deposits	–	IgG+ C1q+	–	–	C1q++	–	–	–	–	IgG++	IgG+++ IgM+ C3+ C1q+	IgA + IgG+++ IgM+ C3+	–	–	IgG+/− C1q+/−
Necroziting vasculitis	–	+	–	–	–	–	–	–	–	–	–	–	–	++	–
Chronic vascular lesions	–	+	+	–	+	+	–	+	+	+	+	+	+	+	+
Vascular Immune deposits	–	IgG+++ IgM+ C3+ C1q++	IgG++ IgM++ C3+ C1q+	–	C1q+	–	–	–	–	–	IgG+++ IgM+ C3+ C1q+	IgA + IgG+++ IgM+ C3+	C3+	C3+/−	C3+

*MPGN* membranous and proliferative glomerulonephritis, *CGN* crescentic glomerulonephritis, − negative, + positive, ++ moderate, +++ abundant

### Treatment and outcome

**Fig. 2:**
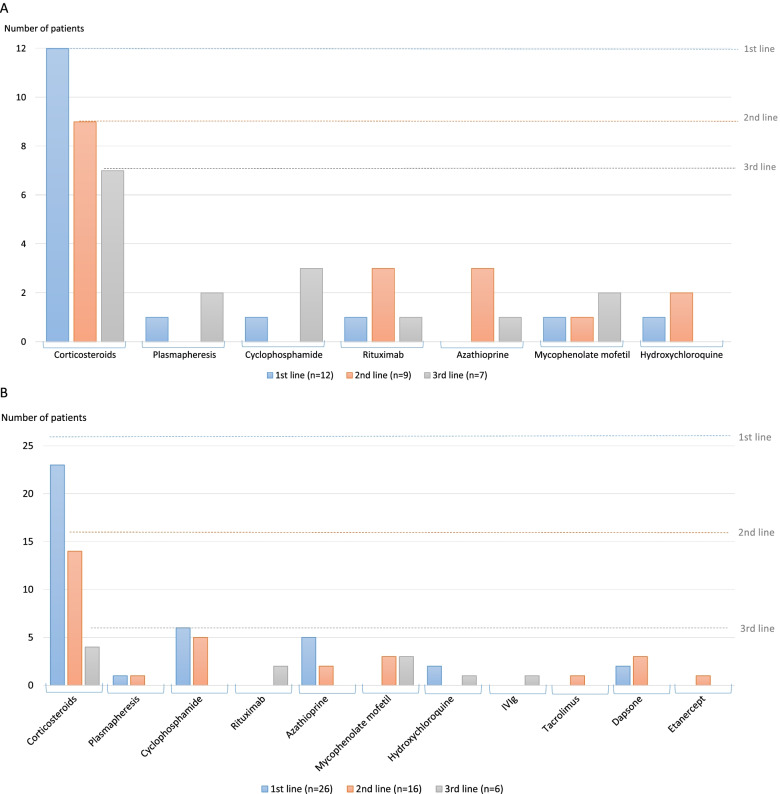
Treatment of the kidney involvement of HUV (first, second and third line therapies). **A** In the 12 patients from the present cohort. **B** In the 26 patients from the literature

**Fig. 3:**
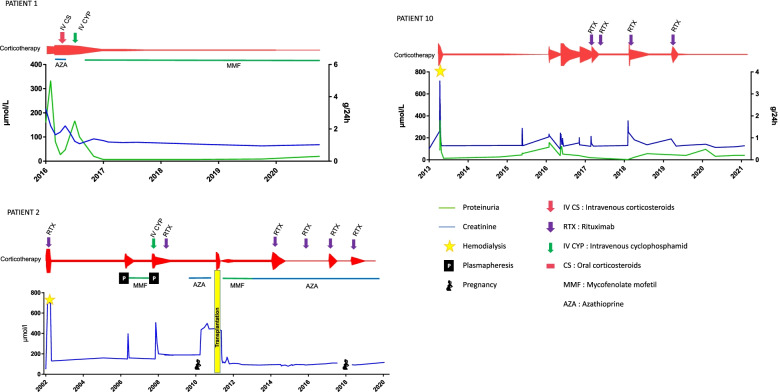
Treatment and renal outcome in 3 patients representative of the present cohort (patients #1, #2 and #10)

Corticosteroids were prescribed in all patients, for all renal flares of HUV, as shown in Fig. 2A. Corticosteroids were tapered and continued for several years in some patients. An additional immunosuppressive therapy was prescribed in 9 patients. Rituximab was used in 5/12 patients (1 as a first line therapy, 3 after corticosteroids, 1 after cyclophosphamide). It allowed a remission in 4 patients (patient #9, with severe AKI initially requiring dialysis, was not in remission at last follow-up), and was reinfused successfully in 2 patients who relapsed. Patients were followed-up for an average of 8.9 years. The detailed evolution of patients is described in Fig. 3 and Supplementary Fig. 1. The total number of renal flares per patient ranged from 1 to 8. An infectious trigger for renal relapses was documented in 2 patients (one with pneumococcal pneumonia, and one with several relapses occurring after viral or bacterial infections). Plasma exchanges were used in 2 patients from this cohort: patient #2, for a relapse of HUV with severe AKI, which required also successively mycophenolate mofetil, cyclophosphamide and rituximab; and patient #9, for persistent severe AKI with massive proteinuria despite corticosteroids and rituximab. Patient #2 reached ESRD 2 years later, after an unplanned pregnancy, and received a kidney transplant without further relapse of HUV. Her second pregnancy was carried-out normally while she was transplanted, without HUV relapse.

At last follow-up, all patients were alive. Patient #2 had received a kidney transplant, patients #3 and #7 were on chronic dialysis, patient #9 had stage 5 CKD, patients #10 had stage 3A CKD. The other 7 patients had a preserved eGFR (≥60 mL/min/1.73m^2^) without proteinuria. A severe complication of immunosuppressive therapy had occurred in 2 patients (one bacterial pneumonia and one cryptococcal meningitis).

We investigated the possible relationship between histological damage and HUV outcome, and the possible predictive factors for prognosis (Supplementary Tables 3, 4 and 5). Only baseline serum creatinine was predictive of CKD (eGFR < 60 mL/min/1.73m^2^) and of relapses.

### Kidney involvement of HUV in the literature

Case reports of kidney involvement of HUV from the literature were collected and compared to patients from the present cohort (Table 3) [9–31]. The clinical presentation of the 26 patients with kidney involvement of HUV from the literature was consistent with the present work but high blood pressure was more frequent at diagnostic in our cohort (*p* = 0.005). The broad range of kidney lesions observed in the present cohort was also observed in the literature. Cryoglobulinemia and ANCAs antibodies were more frequent in our cohort (respectively *p* = 0.002 and 0.02) and the follow-up was longer (*p* = 0.002). Out of these 26 patients, 5 died, 4 reached ESRD and 2 developed stage 3 CKD.

**Table 3:** Comparison of patients from the present cohort with patients with kidney involvement of HUV from the literature

	Patients report	Litterature review	p	Total
Number of patients	**12**	**26**		**38**
Age at diagnosis (years), mean	42	32	0.14	35
Female gender, n (%)	9 (75%)	16 (61%)	0.49	25 (66%)
**Clinical examination**				
Asthenia/fever	10 (83%)	13 (50%)	0.07	23 (60%)
Chronic urticaria	12 (100%)	26 (100%)	1	38 (100%)
Angioedema/purpura/livedo	7 (58%)	13 (50%)	0.73	20 (53%)
Cutaneous leucocytoclastic vasculitis	7/9 (78%)	18/18 (100%)	0.1	25/27 (92%)
Arthralgia/arthritis	8 (67%)	13 (50%)	0.49	21 (55%)
Anterior uveitis/conjunctivitis	4 (33%)	8 (30%)	1	12 (32%)
Retinopathy	2 (17%)	1 (3%)	0.23	3 (8%)
Lymphadenopathy	5 (42%)	5 (19%)	0.23	10 (26%)
PRESS/peripheral neuropathy	2 (17%)	2 (7%)	0.58	4 (11%)
Abdominal pain	1 (8%)	10 (38%)	0.12	11 (29%)
Respiratory disease	3 (25%)	5 (19%)	0.69	8 (21%)
Cardiac involvement	1 (8%)	3 (11%)	1	4 (11%)
**Renal presentation**				
Proteinuria	12 (100%)	22 (84%)	0.29	34 (89%)
Nephrotic syndrom	6 (50%)	7 (26%)	0.27	13 (34%)
Microscopic/macroscopic hematuria	9 (75%)	17 (65%)	0.71	26 (68%)
Acute kidney injury	8 (67%)	10 (38%)	0.16	18 (47%)
Hemodialysis at diagnosis	2 (17%)	1 (3%)	0.23	3 (8%)
High blood pressure	7 (58%)	3 (11%)	**0.005**	9 (24%)
**Biology**				
Elevated CRP, fibrinogen or ESR	5 (42%)	10 (38%)	1	15 (39%)
Low C3	10 (83%)	23 (88%)	0.64	32 (84%)
Low C4	11 (92%)	21 (80%)	0.64	31 (82%)
Low CH50	9 (75%)	14 (53%)	0.29	22 (58%)
Low C1q	7 (58%)	10 (38%)	0.31	17 (45%)
AntiC1q antibody	8 (67%)	10 (38%)	0.16	17 (45%)
Antinuclear antibody	7 (58%)	8 (30%)	0.16	14 (37%)
Antiphospholipid antibody	1 (8%)	1 (3%)	0.54	2 (5%)
Rheumatoid factor	2 (17%)	2 (7%)	0.58	4 (11%)
Cryoglobulinemia	5 (42%)	0	**0.002**	3 (8%)
ANCA	6 (50%)	3 (11%)	**0.02**	8 (21%)
Monoclonal gammopathy	1 (8%)	0	0.32	1 (3%)
**Renal biopsy**				
Membranoproliferative GN	8 (67%)	15 (57%)	0.73	23 (61%)
Membranous nephritis	0	3 (11%)	0.54	3 (8%)
Lupus like GN (diffuse deposits only)	0	5 (19%)	0.16	5 (13%)
Crescentic GN/vasculitis	3 (25%)	2 (7%)	0.30	5 (13%)
Interstitial nephritis	1 (8%)	1 (3%)	0.54	2 (5%)
**Mean duration of follow up (months)**	107	44	**0.002**	71
Deceased	0	5 (19%)	0.16	5 (13%)
End-stage kidney disease	4 (33%)	4 (15%)	0.23	8 (21%)
Transplantation/Dialysis	1 (8%)/2 (17%)	1 (3%)/3 (11%)	0.54/0.54	2 (5%)/5 (13%)
eGFR: 15-60 ml/min/1.73 m2	2 (17%)	3 (11%)	0.64	5 (13%)
Persistant proteinuria (> 0.3 g/24 h or g/g)	4 (33%)	4 (15%)	0.23	8 (21%)

*PRESS* Posterior reversible encephalopathy syndrome, *CRP* C-reactive protein, *ESR* elevated sedimentation rate, *ANCA* anti-neutrophil cytoplasmic antibodies, *GN* glomerulonephritis, *eGFR* estimated glomerular filtration rate

Treatments administered to the patients from the literature are detailed in Fig. 2B. Two patients with kidney involvement received rituximab as a third line therapy: the first patient experienced a full recovery of kidney function after 12 months of dialysis dependence [31], the second was a child without available data on outcome [24]. Plasma exchanges were used in 2 patients from this cohort but were not sufficient and required additional treatment with rituximab or cyclophosphamide. Only 2 patients from the literature were treated with plasma exchanges for AKI (1 reached ESKD, the other reached remission) [13, 22].

Considering both the present cohort and patients from the literature (total *n* = 38), the presence of anti-C1q antibodies was not associated with outcomes: 41% of patients with positive anti-C1q had an unfavourable outcome (ESKD or death) versus 33% of patients without antiC1q (*p* = 0.74).

## Discussion

This is the first study describing specifically biopsy-proven kidney involvement of HUV, with detailed pathological data and long term follow-up. We show that HUV can be associated with different glomerular lesions (mainly MPGN, but also crescentic GN, with or without immune glomerular deposits), lesions of renal arterioles (both acute and chronic, sometimes with C1q and immune complex deposits), and tubulo-interstitial inflammation (sometimes with tubular C1q and immune complex deposits). Notably, at the time of kidney biopsy, the interstitial fibrosis was often absent or mild, suggesting a rapid and recent occurrence of renal lesions. This kidney involvement can be life-threatening, with AKI requiring emergency dialysis, and can lead to ESKD. Yet, even with a severe initial presentation, a complete renal recovery can be obtained with corticosteroids, and possibly immunosuppressive therapy, in a majority of patients.

HUV is an immune-complex mediated systemic vasculitis affecting small vessels [1]. Immune complex deposits in vessel walls are usually documented on skin biopsies of urticarial lesions, and we show here that these deposits, comprising C1q, can also be encountered in renal arterioles.

Yet, immune deposits are not systematic in the kidney of patients with HUV, especially in glomeruli, where pauci-immune forms of crescentic GN can be encountered. An overlap between HUV and ANCA-associated vasculitis (AAV) could be discussed in these patients. Here, among the 6 patients with positive ANCA antibodies, only 4 had specific ANCA, among whom 3 displayed MPGN (classically not encountered in AAV) and only 1 displayed pauci-immune CGN. This last patient, indeed, both responded to HUV classification criteria (chronic urticaria, complement consumption, arthralgia, glomerulonephritis, and anti-C1q antibodies) and displayed features of AAV (arthralgia, glomerulonephritis and anti-MPO antibodies).

Similarly, the possible overlap between HUV and systemic lupus erythematosus (SLE) can be discussed in patients with positive ANA. Among the 7 patients with positive ANA, only 1 had anti-dsDNA, and displayed isolated interstitial nephritis. Only 1 patient with positive ANA displayed a MPGN with full-house immune deposits. Apart from this kidney involvement, this patient had no typical feature of SLE but displayed: biopsy-proven cutaneous leucocytoclastic vasculitis, angioedema, and recurrent bronchitis with chronic respiratory failure.

HUV is a rare disease, but its exact incidence is unknown [6]. Kidney involvement of HUV is even more rare: in the largest retrospective cohort of HUV, comprising 57 cases gathered in France over 20 years, 10 (18%) patients displayed kidney involvement of HUV [5]. In an American cohort comprising 18 patients with HUV, 9 (50%) patients displayed kidney involvement [4]. More recently, a Swedish cohort of 16 patients reported 5 patients with kidney involvement, among whom 2 reached ESKD [6]. MPGN, membranous GN, minimal change disease, focal glomerulopathy and anti-glomerular basement membrane nephritis have been described in the literature [32]. We confirm in this work the heterogeneity of clinical presentation and kidney pathological lesions in patients with HUV.

AntiC1q antibodies are IgG antibodies directed against the collagen-like region of C1q. They are encountered in HUV and in other diseases associated with immune complex deposition, such as mixed connective tissue disorder, SLE and post-infectious vasculitis [5]. The presence of anti-C1q antibodies has been associated with kidney involvement in SLE, and more specifically with crescentic lesions [33]. The presence of anti-C1q antibodies was only a minor criterion in Schwartz et al. [2], but was considered more discriminant in the revised international Chapel Hill consensus conference of 2012 [1]. However, the specificity and sensitivity of anti-C1q antibodies are unknown. Their presence is neither sufficient nor mandatory to diagnose HUV: 67% of patients from the present cohort, and 38% of patients from the literature had positive anti-C1q antibodies. In the French retrospective cohort by Jachiet et al., 55% of HUV patients had positive anti-C1q antibodies and 90% had a low C1q, and 88% from the American cohort by Wisnieski et al. had positive anti-C1q antibodies. The prognostic value of anti-C1q antibodies in HUV is controversial: it was associated with renal involvement in the French cohort by Jachiet et al., but showed no prognostic value for the global outcome (ESKD or death) in the present cohort and in the literature review of patients with kidney involvement.

The treatment of HUV and of its kidney involvement is far from established. While corticosteroids were used in all patients from the present cohort, Rituximab was used mostly as a 2nd or 3rd line therapy, but could be proposed as a corticoid-sparing agent in relapsing forms of HUV.

We underline the value of repeat kidney biopsy in these patients, which can allow the differentiation of active versus chronic renal lesions and guide the need for immunosuppressive regimen.

## Conclusion

Patients with HUV can display heterogeneous kidney lesions with glomerular, vascular and tubulo-interstitial involvement, with or without immune complex deposits. Kidney involvement of HUV can be severe and relapsing, and lead to ESKD. Corticosteroids, and Rituximab in severe forms, could be the preferential therapeutic options. Screening for proteinuria, and monitoring of blood pressure and serum creatinine could be proposed in all patients with HUV followed-up in Rheumatology or Dermatology, at the time of the diagnosis and during subsequent flares, to diagnose and treat kidney lesions early to avoid chronic kidney damage.

## Supplementary Information

**Additional file 1: Supplementary Table 1.** Comparison of patients’ characteristics between those who had positive ANCA at some point during the follow-up, and patients who never had positive ANCA. **Supplementary Table 2.** Comparison of patients’ characteristics between those who had positive anti-nuclear antibodies at some point during the follow-up, and patients who never had positive anti-nuclear antibodies. **Supplementary Table 3.** Comparison of patients’ characteristics between those with a favorable outcome (eGFR ≥30 mL/min/1.73m^2^) at last follow-up and those with an unfavorable outcome (eGFR < 30 mL/min/1.73m^2^). **Supplementary Table 4.** Comparison of patients’ characteristics between those with a favorable outcome (eGFR ≥60 mL/min/1.73m^2^) at last follow-up and those with an unfavorable outcome (eGFR < 60 mL/min/1.73m^2^). **Supplementary Table 5.** Comparison of patients’ characteristics between those who relapsed and those who did not relapse during the follow-up.

**Additional file 2: Figure S1**. Treatment and renal outcome of the 9 other patients from the present cohort.

## Data Availability

The datasets used and/or analysed during the current study are available from the corresponding author on reasonable request.
