# Supplementary material for: Biopsy-proven kidney involvement in hypocomplementemic urticarial vasculitis
Source: BMC Nephrol. 2022 Feb 16;23:67. doi: 10.1186/s12882-022-02689-8 (PMC8851735; doi:10.1186/s12882-022-02689-8)
Supplement: Supplementary file 1 — Additional file 1: Supplementary Table 1. Comparison of patients’ characteristics between those who had positive ANCA at some point during the follow-up, and patients who never had positive ANCA. Supplementary Table 2. Comparison of patients’ characteristics between those who had positive anti-nuclear antibodies at some point during the follow-up, and patients who never had positive anti-nuclear antibodies. Supplementary Table 3. Comparison of patients’ characteristics between those with a favorable outcome (eGFR ≥30 mL/min/1.73m2) at last follow-up and those with an unfavorable outcome (eGFR < 30 mL/min/1.73m2). Supplementary Table 4. Comparison of patients’ characteristics between those with a favorable outcome (eGFR ≥60 mL/min/1.73m2) at last follow-up and those with an unfavorable outcome (eGFR < 60 mL/min/1.73m2). Supplementary Table 5. Comparison of patients’ characteristics between those who relapsed and those who did not relapse during the follow-up. [file 12882_2022_2689_MOESM1_ESM.docx]

**Supplementary Table 1.** Comparison of patients’ characteristics between those who had positive ANCA at some point during the follow-up, and patients who never had positive ANCA.

|  | **ANCA+**  **(n=6)** | **ANCA-**  **(n=6)** | **p** |
| --- | --- | --- | --- |
| Patient N° | 2-7-8  9-11-12 | 1-3-4  5-6-10 |  |
| Baseline characteristics |  |  |  |
| Nephrotic syndrome (%) | 33.3 | 66.6 | 0.54 |
| Serum creatinine, mean (µmol/L) | 279 | 263 | 0.79 |
| Anti-C1q antibodies (%) | 83.3 | 50 | 0.54 |
| **Antinuclear antibodies (%)** | **100** | **16.6** | **0.01** |
| Crescent on biopsy (% of crescents) | 11.6 | 11.6 | 0.93 |
| Crescent on biopsy (Yes/No) | 33.3 | 66.6 | 0.56 |
| Glomerular sclerosis (%) | 15 | 8.3 | 0.78 |
| Glomerular sclerosis (Yes/No) | 50 | 33.3 | 1 |
| Treatments received |  |  |  |
| Rituximab | 50 | 33.3 | 1 |
| Cyclophosphamid | 33.3 | 33.3 | 1 |
| Plasmapheresis | 33.3 | 0 | 0.45 |
| Mycophenolte mofetil | 33.3 | 50 | 1 |
| Azathioprin | 16.6 | 50 | 0.54 |
| Evolution |  |  |  |
| Relapse | 50 | 50 | 1 |
| Unfavorable outcome (eGFR < 30) | 50 | 16.7 | 0.54 |

**Supplementary Table 2.** Comparison of patients’ characteristics between those who had positive anti-nuclear antibodies at some point during the follow-up, and patients who never had positive anti-nuclear antibodies.

|  | **ANA+**  **(n=7)** | **ANA-**  **(n=6)** | **p** |
| --- | --- | --- | --- |
| Patient N° | 2-4-7  8-9-11-12 | 1-3-5  6-10 |  |
| Baseline characteristics |  |  |  |
| Nephrotic syndrome (%) | 42.8 | 60 | 1 |
| Serum creatinine, mean (µmol/L) | 252 | 307 | 0.92 |
| **ANCA (%)** | **85** | **0** | **0.01** |
| Anti-C1q antibodies (%) | 71.4 | 60 | 1 |
| Crescent on biopsy (% of crescents) | 11.6 | 11.6 | 0.93 |
| Crescent on biopsy (Yes/No) | 33.3 | 66.6 | 0.56 |
| Glomerular sclerosis (%) | 15 | 8.3 | 0.78 |
| Glomerular sclerosis (Yes/No) | 50 | 33.3 | 1 |
| Treatments received |  |  |  |
| Rituximab | 12.1 | 11 | 0.93 |
| Cyclophosphamid | 42.8 | 60 | 1 |
| Plasmapheresis | 12.8 | 10 | 1 |
| Mycophenolte mofetil | 42.8 | 40 | 1 |
| Azathioprin | 12.1 | 11 | 0.93 |
| Evolution |  |  |  |
| Relapse | 42.8 | 60 | 1 |
| Unfavorable outcome (eGFR < 30) | 42.8 | 20 | 0.57 |

**Supplementary Table 3.** Comparison of patients’ characteristics between those with a favorable outcome (eGFR ≥ 30 mL/min/1.73m²) at last follow-up and those with an unfavorable outcome (eGFR < 30 mL/min/1.73m²).

|  | **Favorable**  **outcome (n=8)** | **Unfavorable**  **outcome (n=4)** | **p** |
| --- | --- | --- | --- |
| Patient N° | 1-4-5-6  8-10-11-12 | 2-3  7-9 |  |
| Baseline characteristics |  |  |  |
| Nephrotic syndrom (%) | 37.5 | 75 | 0.54 |
| Serum creatinine, mean (µmol/L) | 211 | 378 | 0.1 |
| ANCA (%) | 37.5 | 75 | 0.54 |
| Anti-C1q antibodies (%) | 62.5 | 75 | 1 |
| Antinuclear antibodies (%) | 50 | 75 | 0.57 |
| % of crescentic glomeruli, mean | 10 | 13 | 1 |
| Presence of crescentic glomeruli (%) | 50 | 50 | 1 |
| % of sclerotic glomeruli, mean | 8.1 | 18.7 | 0.7 |
| Presence of sclerotic glomeruli (%) | 37.5 | 50 | 1 |
| Treatments received |  |  |  |
| Rituximab (%) | 37.5 | 50 | 1 |
| IV Cyclophosphamide (%) | 12.5 | 75 | 0.06 |
| Plasmapheresis (%) | 0 | 50 | 0.09 |
| Mycophenolate mofetil (%) | 25 | 75 | 0.22 |
| Azathioprine (%) | 25 | 50 | 0.54 |

**Supplementary Table 4.** Comparison of patients’ characteristics between those with a favorable outcome (eGFR ≥ 60 mL/min/1.73m²) at last follow-up and those with an unfavorable outcome (eGFR < 60 mL/min/1.73m²).

|  | **Favorable**  **outcome (n=7)** | **Unfavorable**  **outcome (n=5)** | **p** |
| --- | --- | --- | --- |
| Patient N° | 1-4-5-6  8-11-12 | 2-3  7-9-10 |  |
| Baseline characteristics |  |  |  |
| Nephrotic syndrome (%) | 42 | 60 | 1 |
| **Serum creatinine, mean (µmol/L)** | **145** | **424** | **0.017** |
| ANCA (%) | 42 | 60 | 1 |
| Anti-C1q antibodies (%) | 71.4 | 60 | 1 |
| Antinuclear antibodies (%) | 57.1 | 60 | 1 |
| % of crescentic glomeruli, mean | 10 | 14 | 0.73 |
| Presence of crescentic glomeruli (%) | 57.1 | 40 | 1 |
| % of sclerotic glomeruli, mean | 6.4 | 19 | 0.31 |
| Presence of sclerotic glomeruli (%) | 42 | 40 | 1 |
| Treatments received |  |  |  |
| Rituximab (%) | 28.5 | 60 | 1 |
| IV Cyclophosphamide (%) | 14.2 | 60 | 0.22 |
| Plasmapheresis (%) | 0 | 40 | 0.15 |
| Mycophenolate mofetil (%) | 28.5 | 60 | 1 |
| Azathioprine (%) | 28.5 | 40 | 1 |

**Supplementary Table 5.** Comparison of patients’ characteristics between those who relapsed and those who did not relapse during the follow-up.

|  | **No relapse**  **(n=6)** | **Relapse**  **(n=6)** | **p** |
| --- | --- | --- | --- |
| Patient N° | 1-4-5  8-11-12 | 2-3-6  7-9-10 |  |
| Baseline characteristics |  |  |  |
| Nephrotic syndrome (%) | 33.3 | 66.6 | 0.56 |
| **Serum creatinine, mean (µmol/L)** | **145** | **353** | **0.01** |
| ANCA (%) | 50 | 50 | 1 |
| Anti-C1q antibodies (%) | 83.3 | 50 | 0.54 |
| Antinuclear antibodies (%) | 66.6 | 50 | 1 |
| % of crescentic glomeruli, mean | 10 | 13.3 | 0.79 |
| Presence of crescentic glomeruli (%) | 33.3 | 66 | 0.56 |
| % of sclerotic glomeruli, mean | 7.5 | 15.8 | 0.59 |
| Presence of sclerotic glomeruli (%) | 33.3 | 50 | 1 |
| Treatments received |  |  |  |
| Rituximab (%) | 33.3 | 50 | 1 |
| IV Cyclophosphamide (%) | 16.6 | 50 | 0.54 |
| Plasmapheresis (%) | 0 | 33.3 | 0.45 |
| Mycophenolate mofetil (%) | 33.3 | 50 | 1 |
| Azathioprine (%) | 33.3 | 33.3 | 1 |
