# Supplementary figures and images for: Biopsy-proven kidney involvement in hypocomplementemic urticarial vasculitis
Source: BMC Nephrol. 2022 Feb 16;23:67. doi: 10.1186/s12882-022-02689-8 (PMC8851735; doi:10.1186/s12882-022-02689-8)

## Slide 1
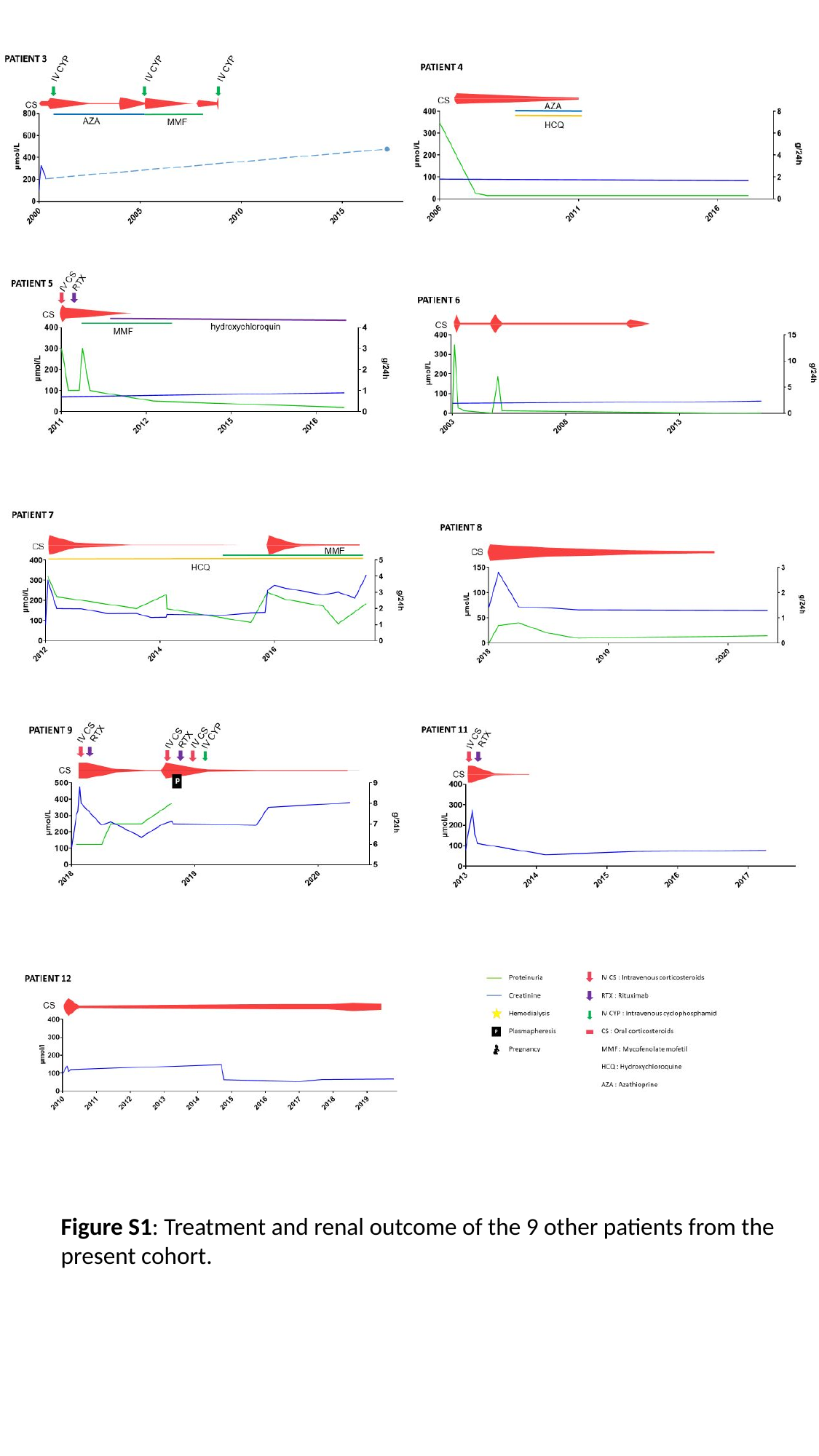

Figure S1: Treatment and renal outcome of the 9 other patients from the present cohort.

Supplement: Supplementary file 2 — Additional file 2: Figure S1. Treatment and renal outcome of the 9 other patients from the present cohort. [file 12882_2022_2689_MOESM2_ESM.pptx]
